# Externally-Triggered Activation and Inhibition of Optical Pulsating Regimes in Quantum-Dot Mode-locked Lasers

**DOI:** 10.1038/s41598-018-30758-2

**Published:** 2018-08-21

**Authors:** Joshua Robertson, Thorsten Ackemann, Luke F. Lester, Antonio Hurtado

**Affiliations:** 1 Institute of Photonics, SUPA Department of Physics, University of Strathclyde, TIC Centre, 99 George Street, Glasgow, G1 1RD UK; 2 SUPA Department of Physics, University of Strathclyde, 107 Rottenrow East, Glasgow, G4 0NG UK; 3 Bradley Department of Electrical and Computer Engineering, Virginia Tech, 302 Whittemore (0111), Blacksburg, VA 24601 USA

## Abstract

Controlled generation and inhibition of externally-triggered picosecond optical pulsating regimes are demonstrated experimentally in a quantum dot mode locked laser (QDMLL) subject to external injection of an amplitude modulated optical signal. This approach also allows full control and repeatability of the time windows of generated picosecond optical pulses; hence permitting to define precisely their temporal duration (from <1 ns spans) and repetition frequency (from sub-Hz to at least hundreds of MHz). The use of a monolithic QDMLL, operating at 1300 nm, provides a system with a very small footprint that is fully compatible with optical telecommunication networks. This offers excellent prospects for use in applications requiring the delivery of ultrashort optical pulses at precise time instants and at tunable rates, such as optical imaging, time-of-flight diagnostics and optical communication systems.

## Introduction

Ultrafast systems utilizing picosecond or femtosecond optical pulses are of increasing importance in a wide range of applications. These expand from high speed electro-optical sampling, all optical clock recovery, material micro machining, THz signal generation and imaging, to name but a few (see for example^1^ and references therein). The high bit rate optical communication capabilities of these ultrafast systems, and their potential in neuromorphic processing^2–4^ is also exciting as we witness the breakdown of Moore’s law and an increasing struggle in microprocessor improvement^5^. Some ultrafast pulsating systems, deemed as ‘pulse-on-demand’ or ‘externally-triggered pulsating’ platforms, are particularly interesting given their capability to controllably switch between continuous-wave (CW) and pulsating modes of operation^6–10^. These switchable systems conveniently offer the functionality of two devices in one and are particularly useful in biological imaging^6^, cell manipulation and cell dissection^7^. For example, switching between CW and pulsating regimes has been obtained with mode-locked solid-state lasers with saturable absorber^8^ and bragg reflector^9,10^ sections. Whilst such systems can provide ultrashort pulses with very high energies, they typically require optical pumping operation, have large footprints, slow repetition rates and yield moderately long switching times (~μs timescales) between CW and pulsating regimes. Hence, new externally-triggered pulsating systems offering reduced footprint, electrical-injection operation and fast switching times between CW and pulsating regimes (<1 ns) are of great interest for use in optical imaging and information and communication technologies.

Semiconductor mode-locked lasers (MLLs) provide ultrashort pulses at repetition rates determined by the laser cavity round trip time^11^ and possess very interesting attributes over solid-state approaches, e.g. compactness, ease of on-chip integration, electrical control, very fast repetition rates (given their reduced cavity lengths) and operation at important telecommunication wavelengths^12–17^. In particular, MLLs based on semiconductor quantum-dash (Q-Dash) and quantum-dot (QD) materials have attracted profuse research effort, as these offer very large bandwidths, lower threshold current densities and excellent temperature performance making them of great interest for use in ultrafast optical systems (see^18,19^ for reviews). In recent years, the technology has developed rapidly and QDMLLs with repetition rates up to hundreds of GHz^20^, yielding ultrashort pulse durations^21,22^ (down to femtosecond regimes), and offering stable mode-locking for wide temperature ranges (up to >100 C) have been reported^23,24^. The effect of external optical injection in QDMLLs has also undergone substantial research effort recently^25–30^. Occurrence of different dynamical regimes as well as bistable operation has been reported in QDMLLs under single and double optical injection schemes^26,27^. Moreover, reduced noise operation, lower pulse jitter, narrower RF linewidths and repetition rate tuning (up to hundreds of MHz) have also been achieved in Q-Dash^31–33^ and QDMLLs^28,29^ by means of external optical injection.

Here, we demonstrate externally-triggered and controllable switching between CW and picosecond optical pulsating regimes in an optically-injected monolithic QDMLL operating at 1300 nm, one of the most important telecom wavelengths. We show that mode-locked picosecond optical pulses (at 5 GHz repetition rates) can be precisely activated or inhibited in a QDMLL under the external injection of intensity modulated optical signals with controlled temporal duration (down to sub-ns lengths). Moreover, this simple technique for externally-triggered pulse control offers very large temporal tunability. Full control over the temporal duration of the periods of activated/inhibited pulsating dynamics and their repetition rates (which can be potentially tuned from sub-Hz to at least hundreds of MHz rates) is demonstrated experimentally. Previous works have reported on the controllable activation/inhibition of spiking dynamics, towards neuromorphic photonic systems, using single-section semiconductor lasers, e.g. vertical-cavity surface-emitting lasers (VCSELs), subject to external optical injection^3,34,35^. However, these demonstrations were not based on mode-locked lasers, but on single-section devices with CW free-running emission (without external perturbations). The operation of such devices relies on fundamentally distinct dynamical responses that do not provide the unique temporal features of the picosecond pulsating mode-locked dynamics of QDMLLs, in terms of ultrashort pulse duration and constant repetition rate. Hence, the present work demonstrates a new, simple yet very powerful, technique for the controllable switching between CW and mode-locked optical pulsating regimes in a QDMLL at very high speeds and with full temporal tunability, while also granting a very small foot-print and operation at telecom wavelengths. This versatile technique offers great potential for future biomedical applications, optical imaging and optical communications and information processing platforms.

## Results

**Figure 1:**
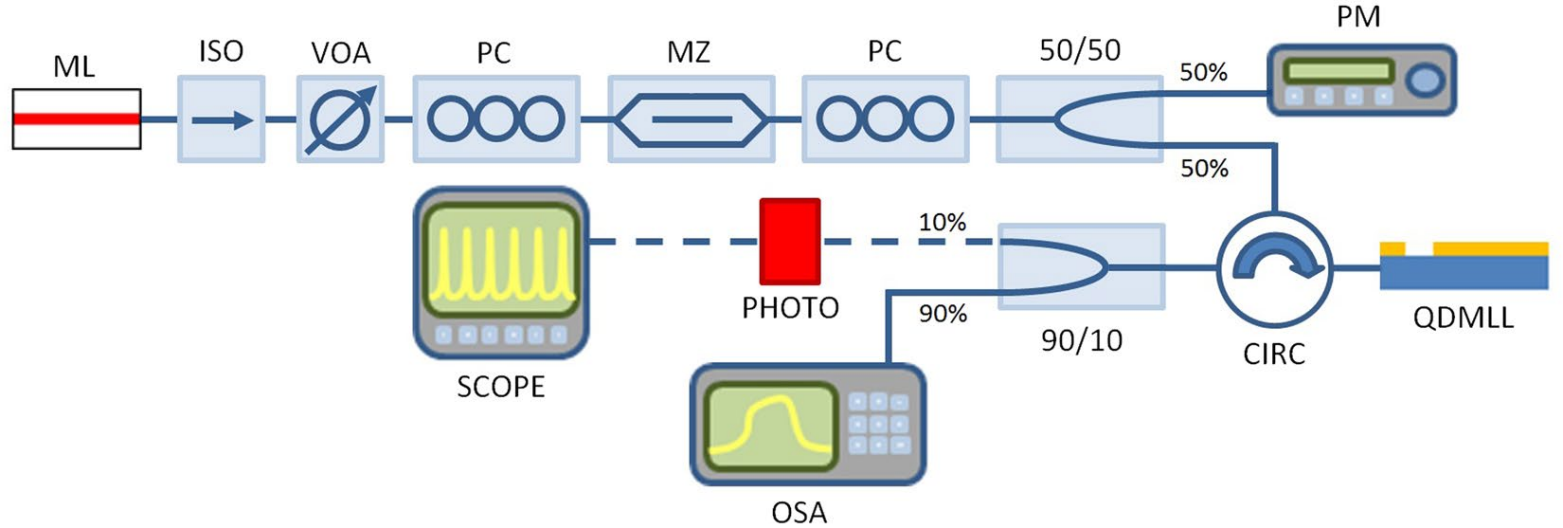
Block diagram of the experimental setup. ML – Master Laser, ISO – Optical Isolator, PC – Polarization Controller, MZ – Mack-Zehnder Modulator, VOA – Variable Optical Attenuator, PM – Power Meter, CIRC – Optical Circulator, QDMLL – Quantum-dot mode-locked laser, PHOTO – Photodiode, OSA – Optical Spectrum Analyser, SCOPE – Oscilloscope.

**Figure 2:**
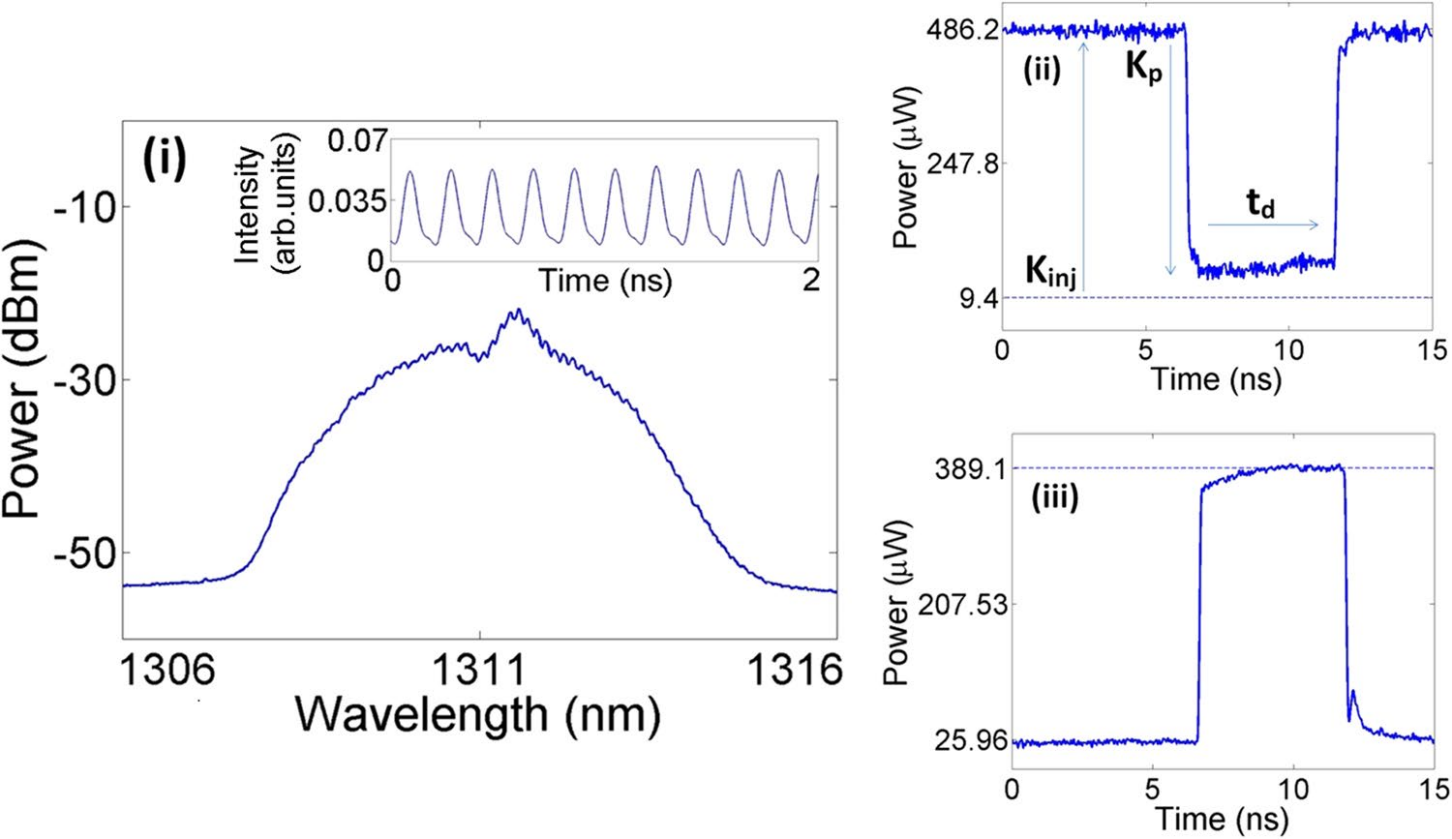
(i) Spectrum and time series of the solitary QDMLL with the device biased at 77.3 mA (gain section), −2.5 V (absorber section) and at 293 K. Time traces of a negative (ii) and a positive (iii) temporal perturbation used respectively to controllably activate and inhibit the mode-locked pulsating devices. (ii) *K* = 0.943 and *t*_*d*_ = 5.15 ns; (iii) *K* = 0.993 and *t*_*d*_ = 5.11 ns.

The experimental setup is shown in Fig. 1. An all-optical fiber system was used to inject externally-modulated light from a tunable 1300 nm laser (Master Laser, ML) into the QDMLL and investigate its temporal and spectral responses. Full details on the experimental setup can be found in the methods section. The QDMLL of this work had an emission wavelength in the 1300 nm range and was designed with a 8-mm long cavity divided into two electrically isolated sections, a saturable absorber and a gain section. The active region of the QDMLL of this work comprised of six layers of InAs Quantum Dots (QDs) embedded in InGaAs Quantum Wells (QWs) and separated by GaAs barriers, in a structure usually referred as Dots-in-a-Well (DWELL). For complete device characteristics, including material growth, QD type, dimensions and features, device fabrication processes, cross-section and epitaxial layer structure see^16,36–38^ and references therein. The device had a threshold current of 56.3 mA (applied to the gain section) at 293 K when the saturable absorber section was driven with a −2.5 V reverse voltage. These values of temperature and reverse bias were kept constant at all times during the experiments. Under these operating conditions, Fig. 2(i) shows that the device exhibited the characteristic mode-locking spectral emission (250 individual modes not resolved), centered at approx. 1311 nm and the measured time series (see inset in Fig. 2(i)) revealed an optical pulsating output with a 5 GHz repetition rate and measured pulse widths of 70 ps (with the limited detection bandwidth available). After deconvolution actual pulse widths from similar devices are determined to be of ~20 ps^16^.

**Figure 3:**
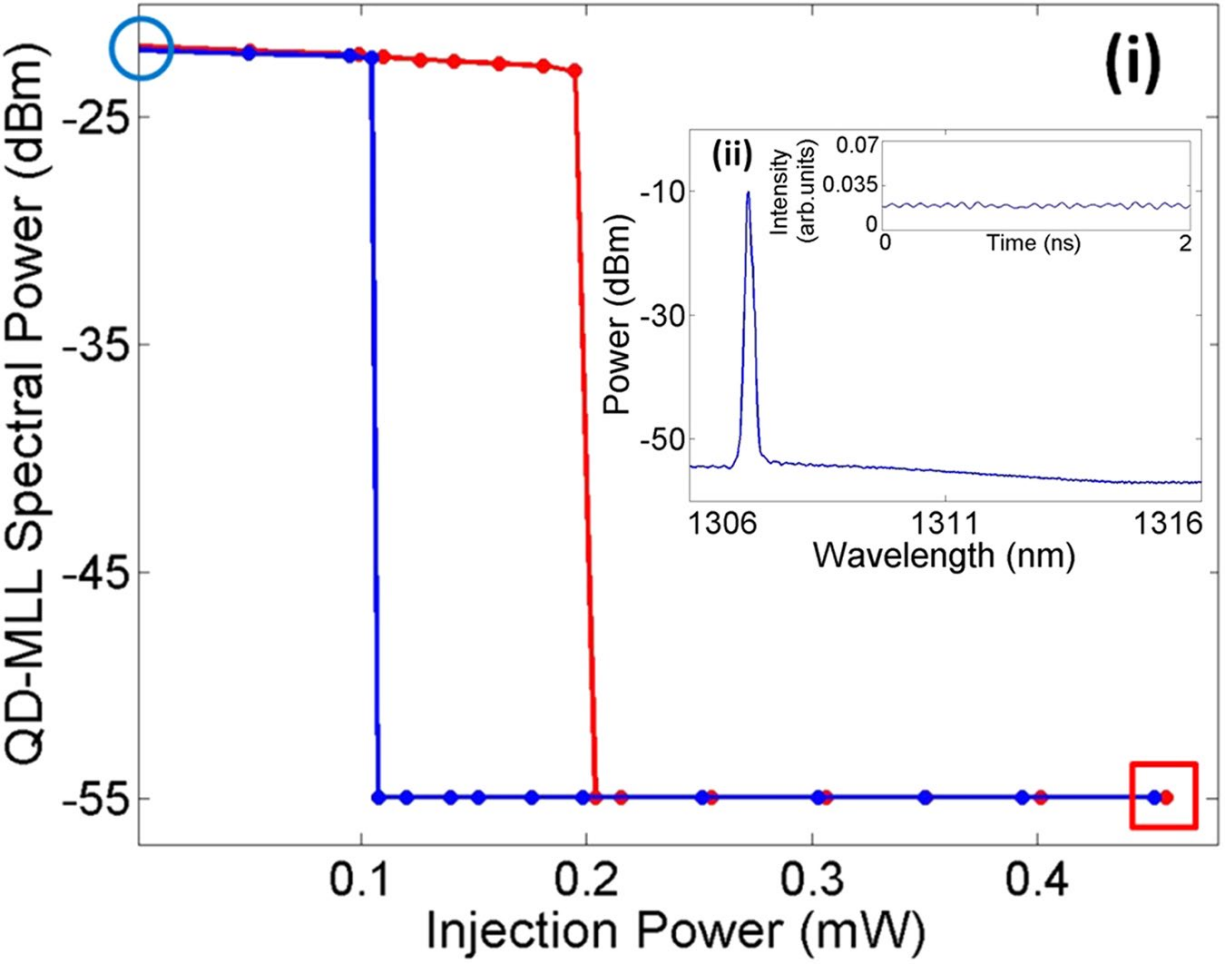
(i) QDMLL’s spectral peak power vs optical input power for increasing (red) and decreasing (blue) injection strength. A plot of the measured spectra (ii) and time series (inset) for the operating point marked by a red square in the curves of (i). The QDMLL is biased with 77.3 mA (gain section), −2.5 V (absorber section) and kept at 293 K. The external optical signal is injected at the wavelength of 1307.18 nm.

We firstly analyzed the static response of the QDMLL under CW external optical injection to determine the system’s different operating regimes. Figure 3(i) plots the relationship between the measured peak power of the QDMLL spectra and the injection strength for increasing (in red) and decreasing (in blue) external optical injection. Figure 3(i) reveals that as the injection power is increased upon a threshold of 203.8 μW, the spectrum of the (solitary) QDMLL spectrum is suppressed and it switches to single-mode emission at the ML’s wavelength. At that threshold value, the ML’s optical power is high enough for the QDMLL to become injection-locked (phase-locked) to the external signal; hence switching its output from mode-locked (wide spectrum and picosecond pulsating output) to stable operation (CW emission and single-peak emission). This situation is kept for increasing values of injection strength above the switching threshold of 203.8 μW. When decreasing the injection strength the reverse process is obtained, and after a threshold in injection strength is overcome the device will no longer be injection-locked to the ML and the power in the (solitary) spectrum of the QDMLL is abruptly recovered. However, the unlocking point is achieved at a lower value of injected power of 104.9 μW yielding a region of bistability with an associated hysteresis cycle. For details on the optical bistable regimes in QDMLLs under external optical injection see^27^).

The static response of the system can be further understood by looking at Fig. 2(i) and in Fig. 3(ii). These show the measured optical spectra and real time-traces captured at the output of the QDMLL for the two points indicated by a blue circle (Fig. 2(i)) and a red square (Fig. 3(ii)) in Fig. 3(i). Figure 2(i) demonstrates that without external optical injection, the solitary QDMLL exhibits mode-locking and a characteristic pulsating output. However, Fig. 3(ii) reveals that under strong enough external optical injection (481 μW), the mode-locked spectrum is suppressed and replaced by a single peak where the QDMLL is injection-locked to the external signal. As a result of the injection-locking the pulsating output is substituted by a constant optical emission characteristic of injection-locking behavior^27^. These results therefore clearly demonstrate that simply by controlling the injection strength the system can be driven either in unlocked or injection-locked regimes with clearly different temporal characteristics. In this work, we will exploit this remarkable yet simple feature to demonstrate a system to controllably activate or suppress the optical pulsating dynamics of the QDMLL (just by simply acting on the injection strength). This system will be able to deliver mode-locked picosecond optical pulses during time windows of precisely-controlled duration and with fully tunable repetition frequencies.

To achieve this goal we have investigated the response of the system to short-temporal perturbations encoded in the optically-injected light from the ML by externally-modulating it. These added perturbations will drive the system at different injection strength values (higher or lower depending on the specific type of perturbation) during their short temporal duration. The two different types of perturbations, shown in Fig. 2(ii) and (iii), are referred to as ‘negative’ and ‘positive’ temporal perturbations. Figure 2(ii) shows first a so-called ‘negative’ perturbation characterized by a higher constant power level (*K*_*inj*_) and a short time interval where the power drops abruptly to a lower level (*K*_*p*_). Figure 2(iii) shows in turn a ‘positive’ perturbation’ with a low power level for most of its duration only interrupted by short temporal intervals where the power is abruptly raised to a higher level. We defined the intensity of the perturbation (*K*) as the ratio between the maximum and minimum power levels (*K* = *K*_*p*_*/K*_*inj*_). These perturbations will cause the system to temporarily switch between the previously described injection-locked and unlocking regimes; hence allowing to controllably activate or suppress the optical pulsating dynamics of the QDMLL. Obviously, providing that these temporal perturbations are configured with sufficient intensity, exceeding the threshold levels to transit between regions of locking/unlocking behaviors (see Fig. 3(i)).

**Figure 4:**
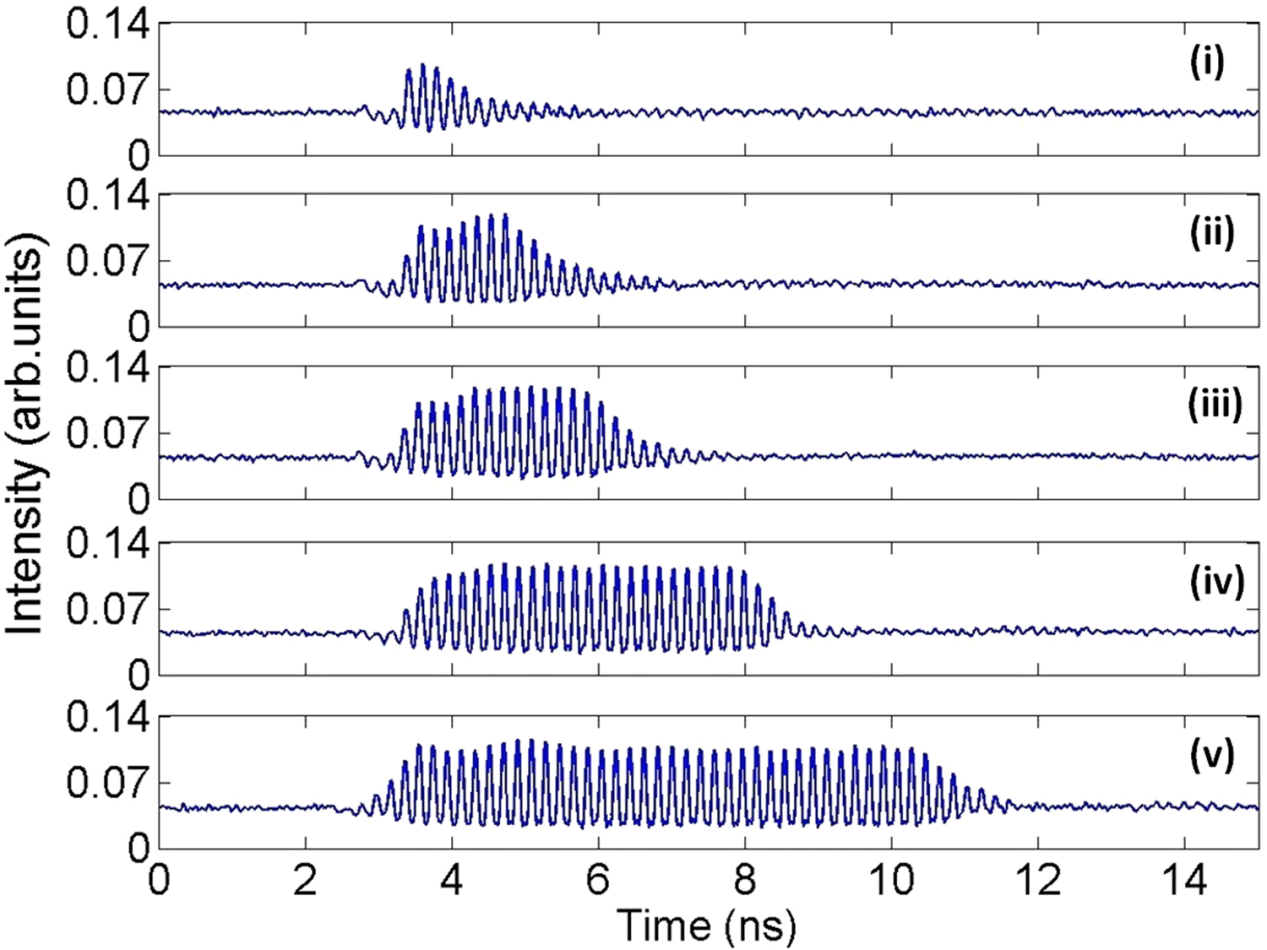
Measured time series at the output of the QDMLL when subject to the optical injection of an external signal with encoded ‘negative’ perturbations with *K* = 0.943 and varying *t*_*d*_: (i) 0.75 ns, (ii) 1.83 ns, (iii) 3.05 ns, (iv) 5.15 ns and (v) 8.02 ns. Controlled windows with tunable temporal length of picosecond optical pulsating regimes are obtained in each case.

We have investigated the response of the system to ‘negative’ or ‘positive’ perturbations (see Fig. 2) to demonstrate both the controllable activation and inhibition of optical pulsating regimes in the QDMLL. Firstly, we considered the case of injecting signals with ‘negative’ perturbations (as in Fig. 2(ii)), encoded in the ML’s light. The QDMLL was driven with the same conditions used for the static analysis of Fig. 3. A current of 77.3 mA and a reverse bias voltage of −2.5 V were applied to the device’s gain and saturable absorber sections respectively. The device’s temperature was also kept constant at 293 K. The external signal was injected at 1307.98 nm and the encoded perturbations had a strength of *K* = 0.943 with a maximum power level of 486.2 μW, repetition rate of 15 MHz and variable yet fully controlled temporal duration (*t*_*d*_). Figure 4 plots the measured real-time traces at the QDMLL’s output under these conditions for increasing values of t_d_ from 0.75 ns to 8.02 ns (as indicated in the caption). For all cases, Fig. 4 demonstrates that bursts of optical pulses with tunable temporal duration (depending on the value of *t*_*d*_) are controllably activated at the output of the QDMLL.

These results can be explained as follows: prior to the arrival of the ‘negative’ perturbation, the initial constant input power level exceeds the threshold value in injection strength for the QDMLL to injection-lock the external signal (see Fig. 3(i)), yielding as a result a constant intensity temporal output. Upon the arrival of the perturbation the sudden reduction in master power, bringing the injection strength below the injection-locking threshold, the QDMLL unlocks from the ML. The system switches to the solitary mode-locked lasing emission triggering the firing of short mode-locked pulses for as long as the perturbation is present. Once the perturbation ends the higher level of optical injection strength is recovered switching back the system from the solitary mode-locking to the injection-locking regime, recovering its initial constant intensity output. Importantly, a fast dynamical response is obtained with mode-locked optical pulses being generated even when very short perturbations of only 0.75 ns (see Fig. 4(i)) are injected into the system. Continuously increasing the temporal length of the perturbation (to values of 8.02 ns) results in the controllable activation of longer bursts of mode-locked pulses from the QDMLL. Notably, fast switching times between the injection locking and mode-locking regimes are obtained, allowing for both the onset and reset transitions between pulsating and constant outputs, with only sub-ns/ns long delays between the arrival/removal of the perturbation and the activation/suppression of the mode-locked pulses. It should be mentioned that as Fig. 4 reveals, the intensity at the leading edge of the bursts builds up over ~0.7 ns before producing the regular mode-locked optical pulsating dynamics. Similarly to the leading edge we observe that upon the removal of the external perturbation, the optical pulses decay over ~1.5 ns until the system recovers the initial injection locking state. We believe that these switching times are due to carrier dynamics in the QDMLL as the system switches between the two regimes, and which we believe are similar to the carrier dynamics between injection-locked and CW regimes in traditional optically-injected semiconductor lasers yielding also similar sub-ns time-scales^3,34,35^. However, we must also note that the measured switching-on/-off times for the pulsating regimes could have been also slightly exacerbated due to the non-zero rise/fall times of the injected perturbations (see Fig. 2). These observed fast switching-on and switching-off times are indeed very encouraging as they outline the potential of this simple system to generate burst of mode-locked pulses with controllable and fully tunable temporal length from sub-ns regimes all the way to seconds and beyond. Analogously, the achieved fast dynamics also suggest the high potential of this system to yield short bursts of mode-locked optical pulses with fully tunable repetition rates (between consecutive bursts) all the way from Hz scales to at least hundreds of MHz without any additional design optimization.

**Figure 5:**
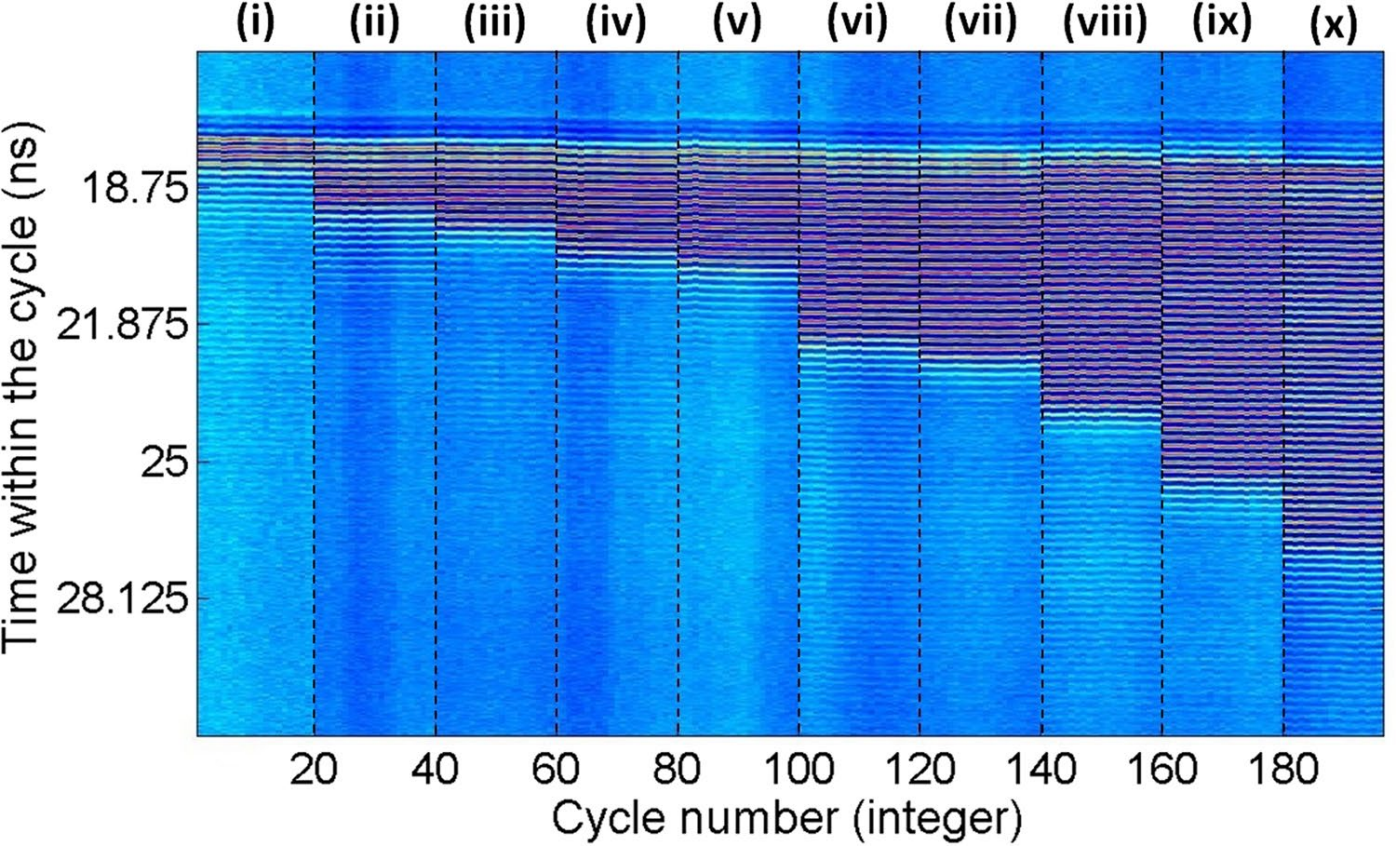
Merged temporal maps obtained for 10 cases of *t*_*d*_: (i) 0.75 ns, (ii) 1.83 ns, (iii) 2.23 ns, (iv) 2.76 ns, (v) 3.05 ns, (vi) 4.79 ns, (vii) 5.15 ns, (viii) 6.42 ns, (ix) 8.02 ns & (x) 9.80 ns. Each map displays the system’s response to 20 consecutive perturbations.

Additionally, to investigate the reproducibility of the achieved results in the generation of bursts of mode-locked pulses, we have produced the temporal map in Fig. 5. This map folds the measured time series in cycles, where the folding parameter is chosen as the time period between two consecutive perturbations. For the results of Fig. 4, obtained at a modulation frequency of *f*_*mod*_ = 15 MHz, is equal to *T* = 1/*f*_*mod*_ = 66.667 ns. This map allows the multiple generated bursts of mode-locked pulses, in response to consecutive perturbations, to be plotted side-by-side for comparison purposes. The temporal map in Fig. 5 merges the response of the system to 20 consecutive perturbations for each of the different values of t_d_ from 0.75 ns to 8.02 ns indicated. The results for each value of t_d_ are separated by dashed lines in the map. Blue/black coloured areas in the map indicate temporal regions of lower intensity (corresponding to the constant intensity regions and pulse), whilst yellow/red areas indicate regions of higher intensity corresponding to pulse peaks in the measured time traces of Fig. 4. Figure 5 clearly reveals the systems’ reproducibility yielding analogous temporally-resolved results for every single perturbation entering the QDMLL, independently of their duration.

**Figure 6:**
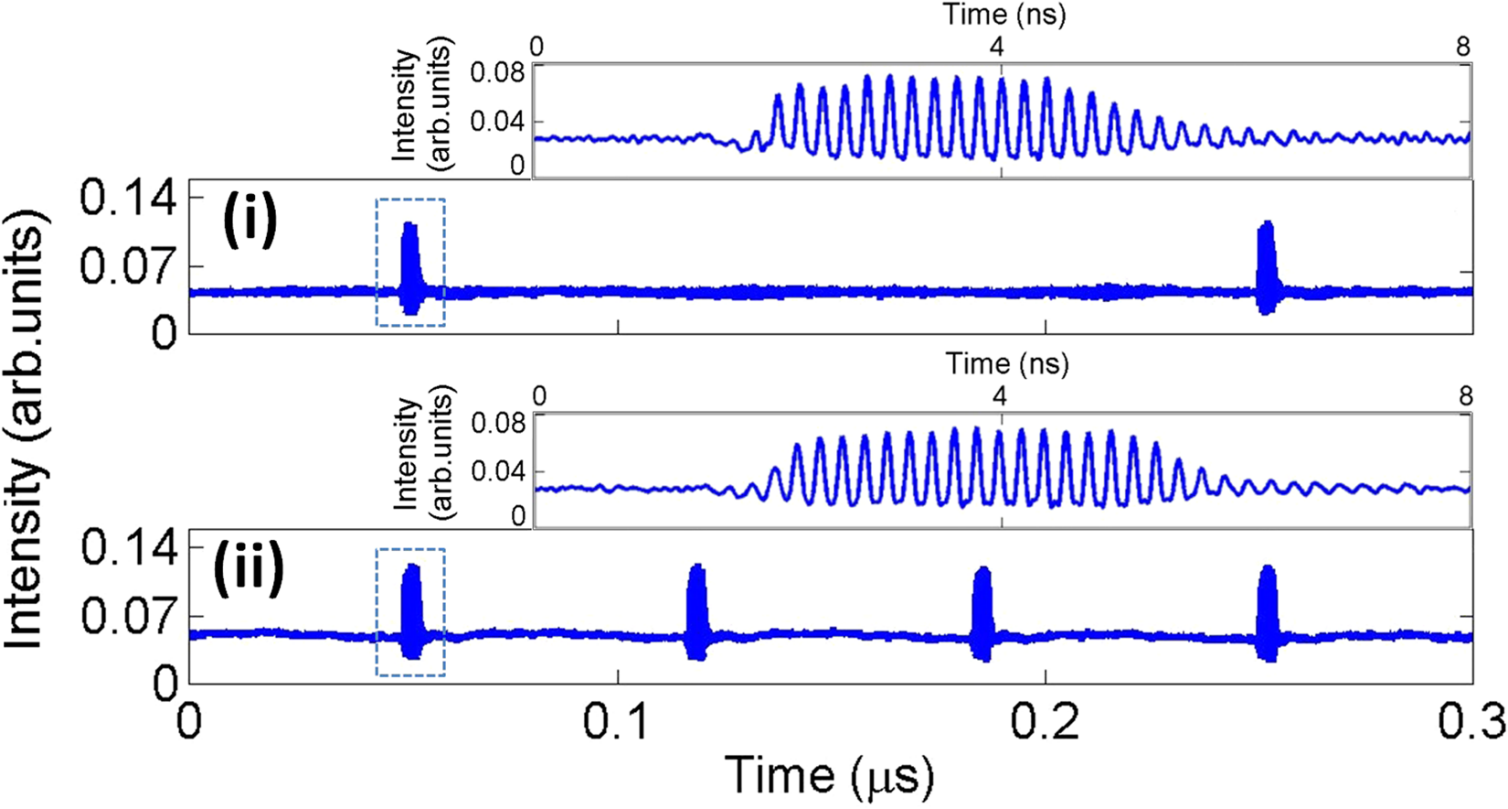
Real-time series measured at the output of the QDMLL showing the controlled generation of 3 ns-long bursts of 70 ps-long (5 GHz repetition rates) mode-locked optical pulses at two different bursting frequencies of 5 MHz (i) and 15 MHz (ii). The insets in the plots show in detail the features of the activated bursts of optical picosecond pulses in each case.

Furthermore, we have also focused on the ability of the reported system to deliver controllable mode-locked optical pulsating regimes with fully tunable repetition frequencies, by simply controlling the modulation frequency of the injected perturbations. Figure 6 provides proof-of-concept of this feature, showing the generation of 3 ns-long bursts of mode-locked optical pulses with two different repetition frequencies of 15 MHz (upper plot) and 5 MHz (lower plot). In both cases, independently of the modulation frequency set, the incoming perturbations produced similar temporal dynamics. The scheme therefore permits the activation of optical picosecond pulses with full control on their burst length and burst frequency. The tunability of the bursting frequency was experimentally demonstrated from frequencies as low as a few Hz to values up to 15 MHz and which were only limited by the capabilities of our experimental setup. However, the short rise and decay times of ~0.7 ns and ~1.5 ns discussed before suggest an extension to at least hundreds of MHz and potentially GHz.

**Figure 7:**
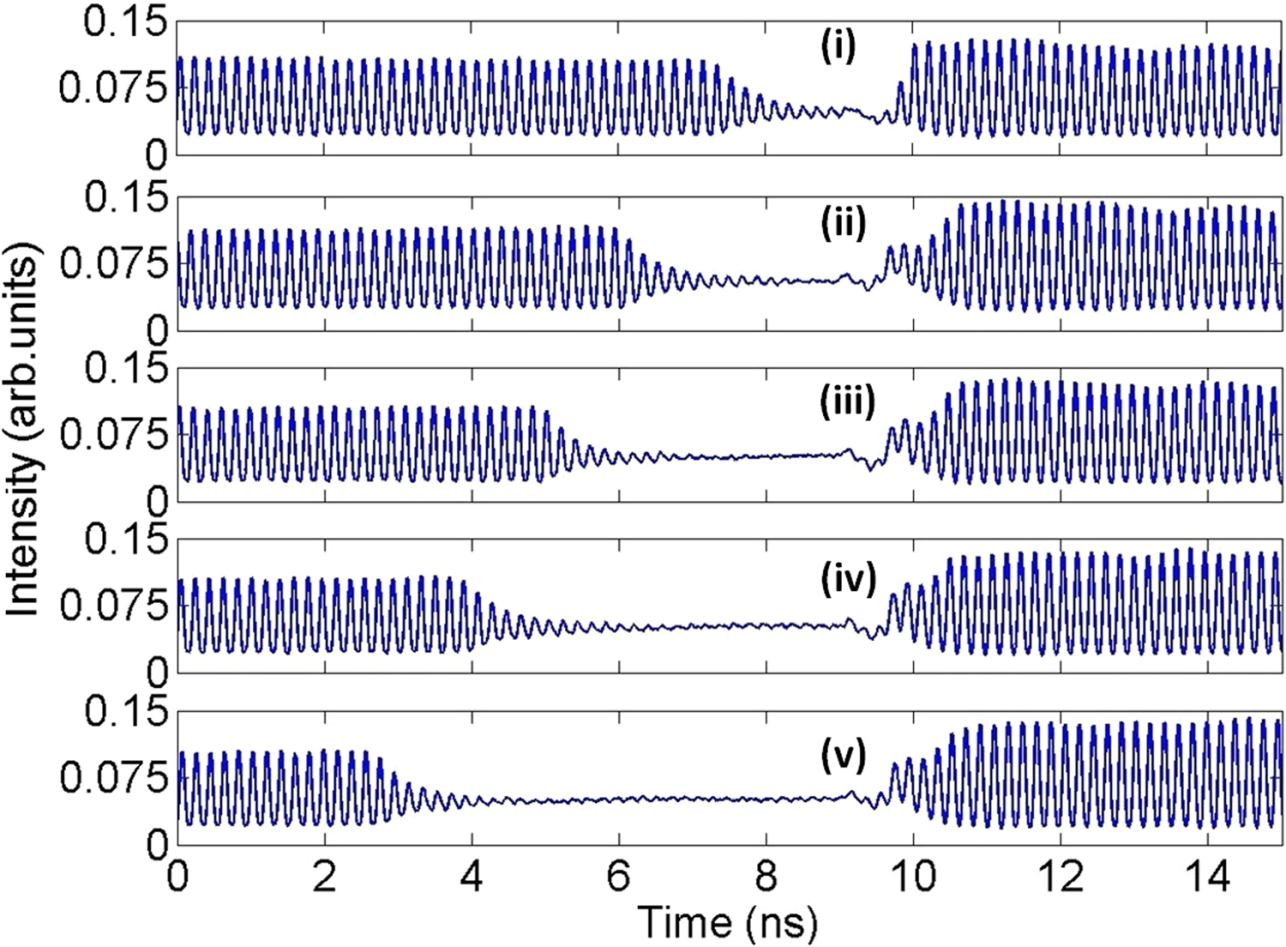
Measured time series at the output of the QDMLL when subject to the optical injection of an external signal with encoded ‘positive’ perturbations with *K* = 0.993 and varying *t*_*d*_: (i) 1.79 ns, (ii) 3.02 ns, (iii) 4.08 ns, (iv) 5.11 ns and (v) 6.36 ns. Controlled windows with tunable temporal length of inhibited picosecond optical pulsating regimes are obtained in each case.

Finally, we have also investigated the potentials of the system to controllably suppress the mode-locked optical pulsating regimes during short specific temporal windows. To do so, we analyzed the response of the 1310 nm-QDMLL of this work to the external injection of modulated optical signals with encoded ‘positive perturbations’, like that included in Fig. 2(iii). The QDMLL was set with the same initial conditions as those used for the experimental findings of Figs 4 and 5. ‘Positive’ temporal perturbations with strength of *K* = 0.933 and different temporal durations from *t*_*d*_ = 1.79 ns to *t*_*d*_ = 6.36 ns were optically injected into the system. The measured real-time traces obtained at the output of the QDMLL in each case are plotted in shown in Fig. 7(i-v).

**Figure 8:**
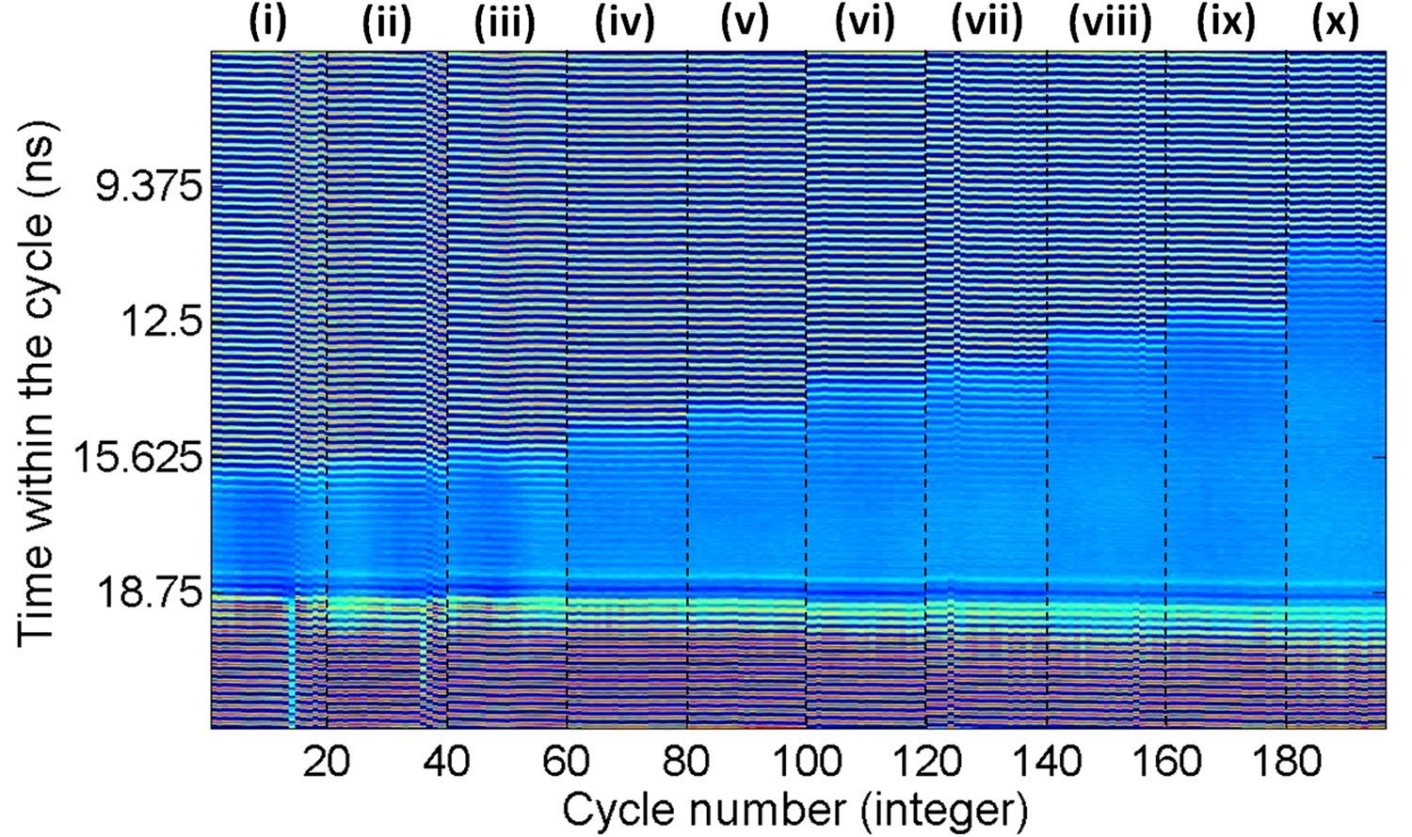
Merged temporal maps obtained for 10 cases of *t*_*d*_: (i) 2.53 ns, (ii) 2.77 ns, (iii) 3.02 ns, (iv) 3.58 ns, (v) 4.08 ns, (vi) 4.73 ns, (vii) 5.11 ns, (viii) 5.88 ns, (ix) 6.36 ns & (x) 8.01 ns. Each map displays the system’s response to 20 consecutive perturbations.

These clearly revealed the controllable temporal suppression of the mode-locking optical pulsating dynamics during the short temporal windows of the entering perturbations. In this case, reverse switching from the solitary mode-locking regime of the QDMLL (obtained when no, or very small, optical injection is applied) to the injection-locked regime (obtained upon the arrival of the perturbation with higher injection strength) is obtained. As a result, Fig. 7 shows the achievement of the reverse process obtained in Fig. 4, with the system’s output switches from a mode-locking optical pulsating to a constant output regime for as long as the perturbation is present. The temporal map in Fig. 8 merges the system’s response to 20 consecutive perturbations for 10 different temporal lengths for the entering perturbations (from 2.53 ns to 8.01 ns as indicated), with the results for each value of *t*_*d*_ separated by dashed black lines. The map in Fig. 8 demonstrates also for this second case the high reproducibility of the periods of suppressed mode-locked optical pulsating obtained for multiple consecutive perturbations. Also, the step-like pattern in the map of Fig. 8 illustrates graphically the full tunability of the temporal windows where the mode-locked optical pulsating dynamics are suppressed just by simply varying the temporal duration of the injected perturbations. Similar switching-on and switchin-off times for the suppression and recovery of the optical mode-locked pulses are obtained here in comparison to those measured when investigating the controllable activiation of mode-locked optical pulses (included in Figs 4 and 5).

## Discussion

In summary, this work demonstrates a simple and versatile technique for the generation and inhibition of externally-triggered picosecond optical pulse regimes with a QDMLL. We show that under external injection of a modulated optical signal, the output of a QDMLL can be controllably switched at high speeds between CW and mode-locked optical pulsating operation. This behavior results from the controllably induced transitions in the QDMLL between regimes of injection locking and unlocking operation, yielding respectively CW and picosecond optical pulsating operation, and occurring for the different amplitude levels configured for a modulated optical signal externally-injected into the device. Moreover, our technique is also repeatable yielding exact temporal features for the picosecond optical pulsating responses produced for multiple consecutive injected signals. It also allows fast operation, as well as full control over the temporal length of the windows of generated/inhibited picosecond pulsating dynamics. Further, it also enables full control over the repetition rates between intervals of picosecond pulsating regimes with expected tunability all the way from sub-Hz to up to GHz frequencies just by controlling the modulation frequency of the externally injected signal. Finally, our results are obtained with a device operating at the important telecom wavelength of 1300 nm, making our approach fully compatible with optical communication systems and networks. In conclusion, this work delivers a simple but powerful technique for the generation of controllable and tunable picosecond optical pulsating regimes, using a monolithic QDMLL with very small footprint and operating at telecom wavelengths. This offers exciting prospects for applications requiring controlled delivery at precise times of ultrashort optical pulses, such as optical imaging, biophotonics, time-of-flight and optical communication systems.

## Methods

### Experimental Setup

An all-optical fiber system was used to inject light from a tunable 1310 nm laser (Master Laser, ML) into a 1310 nm-QDMLL. A variable optical attenuator (VOA), and polarization controllers (PCs) were included in the setup to control respectively the injection strength and polarization of the externally-injected light from the ML. A Mack-Zehnder (MZ) amplitude modulator was used to externally modulate the ML’s light and encode short-temporal perturbations (from a Signal Generator, SG) in the externally injected signal. An optical circulator injects the ML’s light into the 1310 nm-QDMLL and collects its reflected light for temporal and spectral analysis. The temporal dynamics are investigated using a 9.5 GHz amplified photodetector and a high-bandwidth (13 GHz) real-time oscilloscope whilst the spectral response is analysed with an Optical Spectrum Analyser (OSA).

### Static Response

The static response of the 1310 nm-QD-MLL device was firstly analysed. For this purpose, the QDMLL was subject to CW optical injection from the ML. The injection power was subsequently controllably increased and decreased, by acting on the VOA. Optical spectra and real-time traces were captured for different values of controlled injection power. The behavior of the system as a function of the optically-injected power was determined from the relationship between the measured peak power of the QDMLL solitary spectrum versus the injection power (see results in Fig. 3).

### Dynamical Operation

The investigation of the externally-triggered activation and inhibition of the mode-locked pulsating dynamics in the QDMLL was carried out by externally modulating the ML’s injected light with encoded short-temporal perturbations. These were delivered by the SG in the form of electrical pulses with controlled amplitude and temporal duration (from 0.2 ns to 10 ns); and tunable repetition frequency from 1 Hz to 15 MHz. These electrical signals were fed into the RF input of the MZ modulator to encode them into the ML’s light. ‘Positive’ and ‘negative’ temporal perturbations were alternatively generated simply controlling the DC bias voltage driving the MZ modulator. For both cases analyzed, the output of the QDMLL was collected and temporally analysed using a fast 9.5 GHz amplified photodetector and a 13 GHz real time oscilloscope (see results in Figs 4, 6 and 7). 2D temporal maps^3,34,35,39^ merging the results obtained for multiple cases of analysis were also produced to investigate the reproducibility of the externally-triggered activation/inhibition of the pulsating dynamics in the QDMLL (see results in Figs 5 and 8).

## Data Availability

The datasets generated during and/or analysed during the current study are available from the corresponding author on reasonable request.
